# Perceptions of indigenous ugandan men on the use of long acting reversible contraceptives (LARCs) by rural women

**DOI:** 10.1186/s40834-023-00246-8

**Published:** 2023-10-16

**Authors:** Ronald Arineitwe Kibonire, David D. Mphuthi

**Affiliations:** College of Human Sciences, School of Social Sciences, Department of Health Studies, University of South Africa, City of Tshwane, South Africa

**Keywords:** Long-acting reversible methods (LARCs), Beliefs, Perceptions, Indigenous men, Rural women, Strategies

## Abstract

Worldwide, unintended pregnancies remain a critical public health challenge, with 74 million women in low- and middle-income countries getting these pregnancies yearly. The African continent alone contributes about 25% of all unintended pregnancies globally. Even though not all unintended pregnancies are unwanted, they can lead to many health problems for mothers and children, like malnutrition, sickness, neglect, or abuse, as well as maternal and infant morbidities and mortalities. Globally, many women die due to complications related to childbirth, either during or after pregnancy. Contraceptives, especially for long-acting reversible Contraceptive methods (LARCs), are among the best interventions to reduce maternal death. LARCs help the mother delay pregnancy and allow for longer intervals in childbirth spacing. However, utilising LARCs globally and in Uganda remains low because of limited male partner support.

The purpose of this phenomenological qualitative research study was to elicit an understanding of the perceptions and beliefs of rural indigenous Ugandan men towards the use of LARCs by rural women. Ultimately the study designed strategies to enhance the uptake of those methods. Purposive sampling was used to identify 65 participants for focus group interviews and 30 for individual interviews comprising married men aged 20 to 49 years. The study was conducted in the Rubanda and Kiboga Districts of Uganda. The researcher used semi-structured questions for individual and focus group interviews.

The data analysis was done by transcribing the interviews, sorting the field notes, organising, and storing the data, listening to recordings, and reading field notes and interviews to look for patterns related to the perceptions and belief systems. Using the identified patterns, the researcher coded and categorised the data to build themes emerging on the phenomenon.

The study established negative perceptions and belief systems among rural indigenous Ugandan men regarding the use of LARCs by their rural women, which acted as barriers to utilisation. These perceptions included side effects, fears, desires, and cultural and religious beliefs. The study recommends strengthening social and behavioural change communication, strengthening service provision for LARCs, and monitoring and evaluation systems for LARCs. Additionally, policymakers should provide a conducive environment for LARC services provision, and the Ministry of Education and Sports, through health training institutions and universities, should prepare pre-service and in-service healthcare workers to provide LARC services.

**Supplementary Information:**

The online version contains supplementary material available at 10.1186/s40834-023-00246-8.

## Background

### Introduction

Worldwide, unintended pregnancies remain a critical public health challenge, with 74 million women in low- and middle-income countries getting these pregnancies yearly. The African continent alone contributes about 25% of all unintended pregnancies globally [1]. Some of the factors associated with unintended pregnancies include lack of male partner support, non-use of contraceptives, maternal low levels of education levels and poverty [2].

Even though not all unintended pregnancies are unwanted, they can lead to many health problems for both mothers and children, like malnutrition, sickness, neglect, abuse, and maternal and infant mortalities [3]. Other effects of unintended pregnancies include high fertility rates, school dropout leading to low education levels, and the feature unemployment opportunities leading to poverty [4]. Estimates indicate that about 61% of unintended pregnancies result in unsafe abortions, one of the leading causes of maternal mortality and morbidities in low and middle-income countries [5]. The challenges resulting from the effects of unintended pregnancies can last for generations. About 86.8% of unintended pregnancies, especially those in low- and middle-income countries, are due to the non-use of modern contraceptives [6, 7].

According to global estimates, without using modern contraceptive methods, unintended pregnancies could result in 25 million unsafe abortions and 47 000 maternal fatalities annually [8]. The estimates also indicate that in 2020 an estimated 287,000 women globally died from a maternal cause, equivalent to approximately 800 women dying every day from maternal causes, which implies that a woman died roughly every two minutes [9]. Out of the reported global maternal deaths, Sub-Saharan Africa (SSA), where Uganda is located, accounted for 70% of the global deaths, followed by Central and Southern Asia, which accounted for 17% of mortalities [4].

Maternal complications resulting from pregnancy and childbirth contributed up to 75% of all global maternal deaths. These complications arise from severe bleeding (mainly after childbirth), infections (usually after childbirth), high blood pressure during pregnancy (pre-eclampsia and eclampsia), complications from delivery and unsafe abortion [4]. Reducing the Number of unintended pregnancies could prevent about 60% of maternal mortalities and 57% of child death, especially in low and middle-income countries [8].

About 48% of pregnancies in Uganda are unintended, with 60% occurring in teenagers aged 15–19 years. In addition, Uganda still has a high fertility rate of 5.4 [10]. The burden of unsafe abortions is about 60%, most of them resulting from unintended pregnancies [11]. The maternal mortality ratio is 336 per 100,000 live births, one of the highest in [12]. Unsafe abortions resulting from unintended pregnancies are one of the leading causes of maternal mortality and morbidity in Uganda [13].

Kigezi region, South-Western Uganda, where Rubanda district is located, has the second-highest maternal mortality ratio of 541 per 100,000 live births, only second to the Karamoja region with 588 per 100,000 live births [14]. Additionally, in North-Central Uganda where Kiboga, another study district, has a maternal mortality ratio of 410 death per 100,000 live births, higher than the national average ratio of 336 per 100,000 live births [14, 15]. Therefore, preventing unintended pregnancies is one of the critical approaches to reducing maternal death. Reducing unintended pregnancies is achieved by reducing the unmet need for family planning by increasing access to modern contraceptive methods [16]. The available contraceptives include short-term, LARCs and permanent contraceptive methods. The LARCs, the most effective reversible contraceptive methods, include implants, copper-bearing Intra-Uterine devices (IUDs), and Levonorgestrel intrauterine devices [12].

### The situation in Uganda

The uptake of the most cost-effective LARCs in Uganda is generally low, and the usage is estimated to be 21.4%, with implants contributing 17.3% and Intrauterine devices comprising only 4.1% of the family planning method mix [17]. The two study districts, Rubanda and Kiboga, have one of Uganda’s highest maternal mortality ratios and high fertility rates (state these rates if they have been documented). The high mortality ratios are related to complications arising from pregnancies and children’s birth, such as haemorrhage, hypertensive disorders, abortion complications, pregnancy-related sepsis and indirect causes [18]. Using contraceptives, especially, will be cost-effective to avert some of the complications that lead to mortalities with a significant reduction of mortality ratio in the two study districts while at the same time reducing the total fertility ratios [5]. However, the uptake of contraceptive services, including LARCs for the two study districts, remains low compared to the national uptake [5]. Rubanda district has an uptake of LARCs of 14%, with a rural total fertility rate of 4.8, whereas Kiboga has a LARCs uptake of 9.2% and a rural fertility rate of 6.3 [14, 20]. The low uptake of LARCs means that a key intervention effective in preventing some of the causes of maternal morbidity and mortalities is not well utilised in the two study districts. The low uptake of LARC is partly blamed on the opposition by indigenous men in Rubanda and Kiboga due to the perceptions and the belief systems they hold [21], just like in other parts of the world have shown [22]. These perceptions and belief systems could also be making the indigenous men in the two districts not support rural women’s use of the LARCs [23, 24].

Therefore, this study endeavoured to generate an informed understanding of the perceptions and beliefs of rural indigenous Ugandan men in Rubanda and Kiboga districts towards using LARCs and, in lieu thereof, develop strategies to enhance the utilisation of LARCs. Based on this statement of the research problem, the researcher developed the following study methodology.

## Methodology

This study applied the qualitative research method as a suitable approach to understanding the perceptions of indigenous Ugandan men on the use of LARCs by rural women. The qualitative approach also guided the selection of the research design that follows.

### Design

The researcher used a cross-sectional study design that employed constructive phenomenological qualitative data collection. A phenomenology is an approach that seeks to get the life experiences of people and the meanings they derive from those experiences [27].

### The setting of the study

This research was done in two rural districts of Rubanda and Kiboga, located in Uganda’s Kigezi in the South-Western and Central North regions, respectively. These study settings were selected because of their rural locations, low uptake of LARCs, high fertility rates, and high maternal mortality rates. Rubanda district is located in the Kigezi region, which has a LARC adoption rate of 14%, a rural total fertility rate of 4.8, and a maternal mortality rate of 541 fatalities per 100,000 live births. In addition, the Central 2 (also known as Central North) sub-region, where Kiboga district is situated, has a LARC coverage rate of 9.2%, a rural total fertility rate of 6.3 and a maternal mortality rate of 410 deaths per 100,000 live births [14]. Relatedly, the low uptake of contraceptives, especially the most cost-effective LARCs in both regions, is lower than the estimated national rate of 21.4% [27].

### Study population

The study population included indigenous married men between 20 and 49 years living in the Ugandan districts of Rubanda and Kiboga districts. For this study, indegenous men are those born and have lived in the two districts for at least two years before participating in this study. This age group of 20 to 49 years was chosen since the researcher assumed, based on the Uganda Demographic Health Survey of 2016, that most indigenous Ugandan men are sexually active, married or have female sexual partners already, who are likely to be still within their reproductive ages, making them potential clients for LARCs [27].

### Sampling and selection of participants

This study used purposive sampling whereby inclusion criteria were used to select the required sample size [28, 29]. The sample size from the district of Rubanda comprised 31 participants from Nyamweru and Muko sub-counties for both focus group discussions and individual interviews. The participants who were not free to share their views in focus group discussions were given a chance to participate in individual interviews after explaining the procedures for both data collection approaches. The researcher selected the sub-counties because of their rural location and the lowest LARCs used within the two districts of Rubanda and Kiboga. In the Nyamweru sub-county, the researcher did one focus group discussion with ten participants and five participants face-to-face interviews.

On the other hand, in the Muko sub-county, the researcher considered one focus group discussion consisting of ten participants and six face-to-face interviews. The total Number of participants for Rubanda therefore were 31 for both interviews. In Kiboga district, there were a total of 30 participants from two sub-counties of Bukomelo and Dwanilo, of which 20 participants were for the focus group discussions (ten for each sub-county) and ten participants for face-to-face interviews (Five from each sub-county). Therefore, the total Number of participants for the two districts was 61. The data saturation determined the threshold for the Number of interviews, the point when the researcher did not get any new information from the new participants. Therefore, the study’s total sample size comprised 61 from both districts of Rubanda and Kiboga.

### Inclusion criteria

The inclusion criteria for the study included Indigenous born within Rubanda and Kiboga districts or any Ugandan men that had lived in the two respective districts for at least two years prior to this study. Additionally, other inclusion criteria included men who were aged 20–49 years, who were married or had female sexual partners and who consented to take part in the study. Additionally, men willing to be tape-recorded and consented to their good mental state were included in the study.

### Exclusion criteria

This study excluded non-indigenous Ugandan men who were unmarried or without female sexual partners and were outside the age bracket of 20–49 years. More to that, men who were not residents of the Rubanda and Kiboga districts for at least two years and those that refused to sign a consent form. Two years of residence was considered because the time was adequate for those men to have interacted with the indigenous men in their sub-counties of residence and were likely to have similar beliefs and perceptions in many aspects, including on LARCs. Additionally, men who did not accept to be recorded and those who were identified as unstable mentally by their local leaders and verified by the researcher well were excluded.

### Data collection method

The researcher used phenomenological data collection methods; focus group and individual interviews were conducted using an open-ended guide with semi-structured questions for face-to-face, in-depth individual interviews and focus group interviews. The interview guides for focus group and individual interviews were different as required by the ethics committees at the University of South Africa and The AIDS Support Organisation but had similar questions. The ethics committees and the Uganda National Council for Science and Technology approved all the data collection tools. The researcher used focus groups and individual interviews to provide alternatives to men who did not want to share their views in the presence of other men in a focus group.

Participants were asked questions to elicit a wealth of information. Data collection was conducted in a natural setting, specifically in an open area near the trading Centres and in community meeting venues where non-participants would not hear what was discussed as arranged by the respective village leaders. The questions in interview tools were translated into Rukiga and Luganda, the two languages spoken in the Rubanda and Kiboga districts, respectively. The interviews were taped and eventually transcribed [30–32].

### Development of data collection tools

Before data collection, tools were developed. These included open-ended focus group and individual interview guides, an information sheet for consent and an informed consent form. The open-ended interview guides allowed the participants to speak freely on the subjects they believed were essential to them, using their own words and articulating their experiences in depth using stories, narratives, and examples. Open-ended questions are a popular choice for individual studies or sections of a questionnaire that require participants to provide candid, personal feedback [33].

After developing research tools, the researcher translated them using local language experts into Rukiga and Luganda, the two languages spoken in Rubanda and Kiboga districts, respectively, for easy understanding by the study participants and research assistants. The English and translated versions of the developed questionnaires were authorised for use by the ethical committee at the University of South Africa under registration number UNISA Rec-240816-052, The AIDS Support Organisation (TASO) ethics committee in Uganda with registration number TASO-2021-56, and final clearance was provided by the Uganda National Council of Science and Technology (UNCST) with the reference number HS2152ES.

### Approval for data collection

To recruit participants, the researcher first got a letter from the Uganda National Council for Science and Technology and the President’s Office authorising the researcher to proceed with data collection in the respective districts. The researcher proceeded with the letter to request permission from the Resident District Commissioners (RDCs) of Rubanda and Kiboga to conduct research in their areas. This permission from the RDCs was significant for security clearance, as they are the heads of security in the districts in Uganda.

After getting permission from the RDCs, the researcher proceeded to the District Health Offices in the two districts to inform the District Health Officers about the study, which was health-related and needed permission at that level. The researcher then proceeded to Muko and Nyamweru Sub-counties in Rubanda district and Bukomero and Dwaniro sub-counties in Kiboga districts for another level of authorisation to move to the village level since they supervise the activities of the villages in those sub-counties, including security and health issues. While in the four sub-counties, the researcher sought permission from the Sub-County local council and the indigenous leaders.

The permission of local council leadership and indigenous leaders was key since they are responsible for monitoring all activities in their respective villages. The village local council chairpersons and the indigenous leaders also helped identify the participants, guided by the inclusion criteria. The local council leaders provided the registers of homes with potential participants, and the researcher selected the homes randomly from the lists provided. This was done to minimise bias by the local leaders in choosing the participants. In addition, the village council leaders assisted the researcher in securing appropriate venues where the individual interviews and focus group interviews took place, under the tree shade in a playground or in open, secure spaces in trading Centres and community halls within the respective villages.

### Interview process

The local indigenous leaders from four villages mobilised the potential participants in Rubanda and Kiboga districts. Ethics issues were re-iterated in the information sheet written in Rukiga and Luganda languages. Participants who consented in writing to participate in the study were considered for individual and focus group interviews. For both districts, the total number of participants was 61 (40 for focus group discussions and 21 for individual interviews), and this sample size was determined by data saturation, the point where no new views were coming from the additional participants. The focus group interview lasted 60 to 80 min, while individual interviews lasted 40 to 50 min. The data collection exercise lasted for 21 days.

The researcher conducted interviews in Rukiga and Luganda, the two languages spoken in the Rubanda and Kiboga districts, respectively. The researcher asked probing questions to keep up the conversation and elicit specific information about the topic. The probing inquiries shed more light on the phenomenon of contraceptives, perceptions, and beliefs regarding LARCs. The researcher offered respondents an opportunity to provide more information on the topic at hand. When the researcher established that no further information emerged from the participants, he appreciated their participation. Individual and focus group interviews were recorded after getting consent from the study participants. The researcher also pledged to the participants to share the findings once the study was complete.

### Data analysis

Individual and focus group interview recordings were transcribed into text format for this study by attentively and repeatedly listening to the interviews in a distraction-free, silent environment. The researcher meticulously transcribed every detail of the recorded interviews [34]. Following transcription, the researcher performed memoing, a method for keeping notes of what was learned from the data [35]. This was done to keep track of the concepts and their relationships within the data to direct the creation of codes and themes. After memoing, the researcher manually coded the data, verifying themes, concepts, and categories before labelling analogous text segments [36, 37].

As a co-coder, an independent researcher from one of the organisations conducting research in Uganda was also utilised. The researcher discussed with the co-coder and reached a consensus on the theme, two categories and nine sub-categories considered for this study. The Theories of Planned Behavior (TPB) and Reasoned Action (TRA), which were this study’s theoretical frameworks, were utilised to direct data analysis by providing structure, themes, and sub-themes. Guided by these theories, the researcher used some components to develop the themes. The components of the theory the researcher used include the theory’s background information, beliefs, and perception components. These components guided the research in developing the three themes during the data analysis. The three themes include the understanding of family planning, which is related to knowledge under information in the background factors of the theories, and the perceptions and belief systems that also directly appear in the theories. The intention component of the theories reflects the men’s support for using LARCs by rural women. The researcher ensured rigour throughout the study process to achieve trustworthiness.

## Results

**Table 1:** Summary of the theme, categories and sub-categories emerging from both focus group and individual interviews for Rubanda and Kiboga Districts

Themes	Categories	Sub-categories
**Perception of LARCs**	***Associated or perceived side effects***	*Prolonged bleeding* *Low libido* *Effect on body organs* *Infertility*
	***Fears***	*Separation and single mothers* *Adultery* *Challenges with removal or management of side effects* *Fear that land could be usurped by other tribes.* *Complications in subsequent deliveries*

In this part, the following abbreviations below are used.

### FGD

Focus Group discussion.

### I

Mean Individual interviews.

### R

Refers to participants from Rubanda District, Nyamweru Sub-County.

### RM

Means participant from Rubanda District, Muko Sub-County.

### K

Refers to Participants from Kiboga District, Bukomero Sub-County.

### KD

Means participant from Kiboga District, Dwanilo Sub-County.

### Perception of LARCs

The study findings revealed that all participants had negative perceptions that were a barrier to the utilisation of LARCs by their rural women. These perceptions included:

### Prolonged vaginal bleeding

Prolonged vaginal bleeding means monthly periods associated with prolonged bleeding or an extremely heavy period that lasts over seven days. Participants indicated that LARCs cause continuous vaginal bleeding among women that use them and submitted this as one of the reasons they did not support their partners using LARCs. In the following quotations, participants gave their responses.

*“When our women use an implant or a capsule (IUD), they make them bleed nonstop. As a man, you can imagine what we feel spending a long time without touching your wife. I cannot allow my wife to use family planning method” (FGD R 7).*

*I have heard that women who use capsules bleed nonstop, making them very weak and unable to work at home and in the garden. Therefore, to avoid my wife getting such problems, I cannot allow her to use an implant (I RM 9).*

*“A woman using a family planning method does not see the monthly periods; when they finally come, she will bleed for all the months she missed. This kind of bleeding can kill her; therefore, I cannot allow my wife to use a LARC method (I K15)”.*

*“Family planning users can bleed profusely and become weak over a long period, and as a man, you find you are starved” (FGD-KD 9).*

### Low libido

The findings from the districts of Rubanda and Kiboga confirmed that men perceive LARCs as a method that reduces sexual strength in both men and women. Participants indicated that LARCs reduce women’s sexual desires, resulting in less frequent sexual activity with their spouses. In other instances, the libido of certain males is diminished. Most participants from both districts refused to embrace the use of LARCs by their women due to the perceived loss of libido. The following quotations represent the responses of some respondents.

*“The LARCs make our women impotent and unresponsive when aroused in preparation for doing the adult game of the bed” (FGD-R1).*

*“When a woman is using the capsule in the arm and in the womb, she becomes less interested in sexual intercourse and when you touch her, it is as if she is a log in bed” (I-K11).*

*“I have heard from other men’s experiences that when a woman uses family planning, their husband’s libido decreases also. I also experienced the same when my wife used a family planning method as she lost interest in sex which made me also to lose sexual urge. Therefore, for this reason, I cannot allow my wife to use any family planning method” (I-R 14)*.

### Effect on the body organs

Under this sub-category, it became apparent that several men hold that LARCs affect both women’s and their own organs. The participants reported that the use of LARCs reduces the size of women’s reproductive organs to the point where they are unable to penetrate during sexual encounters. In addition, they indicated that LARCs cause their genitalia to become smaller, weakened, and unable to please their women.

“*When my wife used a coil in the arm, her womanhood became very small, and I could not enter even after taking a long time trying to stimulate her*” (I RM11).

The participants in this study also believed that when a woman uses implants or IUDs, the methods can disappear into the stomach and heart, which could lead to additional health issues such as malignancies, “pressure” (hypertension), and other complications in the body. The following are excerpts from selected participants.

*“We have heard some women using the capsule in the arm which disappears and ends up in the heart where it causes heart diseases and pressure” (I-K15).*

*“Some of the women who use a capsule that is put in the womb end up causing swellings in the stomach and cancer” (FGD-R10)*.

*“Family planning makes women who use it very weak and yet they have a lot of work to do at home. It also makes women become regularly sick“(FGD-RM 7)*.

### Infertility of women

Infertility is the inability to conceive and produce a child and can affect men or women. A majority of men in this study believe that LARC use results in a delayed return to fertility or lifelong infertility. Therefore, the majority of men favoured short-term or natural methods of contraception over LARCs. Listed below are some of the submissions made by men within this sub-category.

*“Some women take longer to get pregnant when the period of using the long-term family method is done which is not good when a man wants another child” (FGD-KD1).*

*“Women who use long-acting family planning do not produce again when they stop the method because all the eggs in a woman are already destroyed. This is bad when they still have few children making family planning bad” (FGD-R1).*

*“Child may die and if there is a need to produce another one and the woman has been on a long-acting family planning it may be hard to reverse the process” (I KD15)*.

### Domestic violence, separation of couples and single mothers

Participants thought the LARCs might lead couples to separate due to domestic violence, particularly when the woman used the method without her husband’s consent. This is echoed in the following submission: *“A woman using family planning (LARCs) becomes less interested in having a sexual meeting with her husband and when a man tries to have sex by force, a fight erupts, and the woman ends up separating from the husband to become a single mother” (I-K 13).*

Some participants believed using LARCs contributes to reduced libido in women, and when men seek sex, they decline. As a result, the men may leave those women and seek solace with other women, leaving them as single mothers. The following quotation captures this sentiment: *“When your woman is using a family planning method that takes long in the body, her power in bed reduces, and this can cause a man to look for another woman leading to separation of the couple” (FGD KD 3).*

Participants stated that they would prefer their women use short-term and natural contraceptive methods over LARCs to avoid such a situation.

Some participants thought that when women use LARCs, they have very few children, and if they disagree with their spouses, they leave the relationship. This is because they are still attractive to other men. After all, they have not had many children, allowing them to remarry quickly. Participants were against their spouses using LARCs for this reason. This assertion was expressed in Kiboga District but not in Rubanda District.

*“Women who use long-acting family planning may leave marriages because they don’t have many children and are still attractive to other men” (FGD K4).*

*“A woman easily leaves the marriage if they don’t have many children with the man. Therefore, if I allow my wife to use family planning, we will only have a few children, and in case of any misunderstanding, she can easily run away from me. In order to protect my marriage, I cannot allow my wife to use a long-acting family planning method” (FGD K5)*.

### Challenges with removal or management of side effects

Some participants expressed concern about their wives using LARCs because they are inserted for free at health facilities. Still, when the time for removal comes, or their wives get challenges with this method, they are not helped at the same health facilities. They confirmed that the health providers at the health facilities tell them to wait for people (organisations) that inserted them. This is also true for women who want to remove the LARCs. As a result, men indicated spending a lot of money to get their women managed for side effects and removal of the devices. The quotation below reflects some sentiments expressed by participants.

*“When they are calling women to come for family planning to the health centres, they tell them that the services are free, but when they get problems, and they want the methods removed, they are asked a lot of money or referred to places which require payment” (FGD K 5).*

*“I cannot accept that my wife uses a capsule in the arm because when the time for removing them comes, the health workers at the health centre cannot help as they want money” (I-RM 4).*

*“Usually, women who get family planning services get them for free from Marie Stopes, but when they get problems and want to remove them, they cannot be helped by the nearby health facility workers at the public health centres. We spend a lot to remove them from private clinics” (FGD-RM 5).*

### LARCs use leads to adultery

Men believe that if their spouses use LARCs, they are unlikely to be impregnated as a result of extramarital sexual relations, and this will encourage them into such relationships, according to the findings of this study. Therefore, participants from the Rubanda and Kiboga districts believed that LARCs could result in infidelity among women. The same assertion holds for men who believe that, once their partners begin using LARCs, their diminished libido will cause them to seek satisfaction from other attractive women. According to the participants, this has led both men and women to contract sexually transmitted diseases, such as HIV/AIDS.

*“Because the woman knows that she cannot be impregnated by another man when she goes out while using a long-term family planning method, this encourages her to continue cheating” (FGD R4).*

*“Women using a long-acting family planning method tend to be cheaters because they are not worried about being impregnated by other men. So, family planning encourages cheating, and I do not support it to save my marriage” (FGD RM 8).*

*“A woman who is using a family planning method, especially those that take long, will go out for other men because she knows she will not get pregnant since she is protected” (FGD-KD 1).*

*“A woman using a long-term family planning method is very tricky; she can easily sleep with other men as she is sure she can never get pregnant. For this reason, I can never allow my wife to use such family planning methods. Natural ones are better for her” (I-K14).*

### Fear of losing their land to other tribes

Some participants in Kiboga District thought the Ugandan government was encouraging the Baganda to use LARCs so that they would have fewer children. In contrast, other ethnic groups in the same district continued producing without restrictions, according to the same participants. They were concerned that non-Baganda tribes, particularly those from the West (Banyakore, Bakiga, and Banyarwanda), would seize their land and wealth if their tribe had a small population. Some participants reported that numerous organisations were promoting family planning in central Uganda, where Kiboga is located, and they believed there were evil schemes to reduce the Baganda population in Uganda. Therefore, some Baganda in Kiboga District oppose the use of LARCs so that they can maintain a large population. Some of the quotations are stated below.

*“You see the problem? They are telling us Baganda to use family planning (LARCs) when the Banyankole and Banyarwanda are busy producing as many children as possible. They will take our land as many non-Baganda already own a huge chunk of land in our district” (FGD-K7).*

*“Why do they want us to use a long-term type of family planning (LARCs) when other tribes in our sub-county are producing like rabbits? Don’t you think there is a motive for stealing our land, as it is already happening? We cannot support such family planning methods” (FGD-KD 5).*

*“The population of Baganda is still small, and therefore no need to use family planning, especially those methods that work for a long time. As a true Muganda, I must produce as many children as possible since our culture allows and encourages it” (I-K15).*

### LARC use leads to producing children with disability

According to the findings from both the Rubanda and Kiboga districts, participants perceived that LARC use could cause their spouses to give birth to children with disabilities. They claimed that some women who use LARCs have abnormal and disabled children. Consequently, they stated they could not support using LARCs because they want healthy children in their families and communities.

*“As a man, I cannot allow my wife to use long-term family planning because when she is to produce again, she may end up producing a disabled child” (FGD-RM 16).*

*“Some women who use a coil and capsules produce lame babies, and therefore I cannot encourage my wife to use them” (I-KD 14)*.

*“Long-acting family planning is not good as they cause disability. Therefore, as a man, I encourage my wife to use natural family planning to avoid problems of such children who are difficult to care for” (FGD-R8).*

### Complications in subsequent deliveries

The Rubanda and Kiboga Districts participants indicated they feared that when their wives use long-acting contraceptives, they could lead to complications in the subsequent deliveries. Participants suggested that pregnancy complications from LARC use often lead to women getting operated on during delivery or even other complications like excessive bleeding before or after delivery. Participants thought that for women who use LARCs, it becomes hard to push a baby, usually through the vagina, explaining the many caesarian sections where men indicated spending much money to have the operation done. Below are some sentiments from selected participants in the Rubanda and Kiboga Districts.

*“I have heard that family planning causes women to bleed a lot, which means there will be complications to subsequent pregnancies. Therefore, my wife cannot use those methods to avoid feature complications in her other pregnancies” (FGD-K4).*

*“The capsules and coils can lead to complications in the succeeding pregnancies, producing weak babies who eventually die. So, allowing my wife to use such contraceptives is out as a way of preventing feature problems of producing” (FGD-RM 9)*.

*“My friend told me that women who use long-acting family planning end up getting complications in the next pregnancies and end up being operated on; they become weak and can never function normally at home” (FGD-RM 15).*

*“My wife used a family planning method and ended up getting problems during labour after stopping the method. She ended up getting operated on where I spent a lot of money” (I-KD 15).*

### Discussion of the study findings

The study found that indigenous Ugandan men have negative perceptions that retard support for using LARCs by their rural women. These perceptions include Low libido, effects on the body’s organs, infertility, fear of partner separation leading to single mothers, adultery, and worry that other non-Baganda tribes might seize their land are just a few of these perceptions and fear of producing children with disabilities after using the LARCs.

In this section, the discussions for the study findings are presented in the following paragraphs.

### Prolonged vaginal bleeding

These findings are consistent with a study in China by Luo et al. [38, 39], which found irregular vaginal bleeding as a solid barrier to LARCs uptake as men thought it would leave them starved of sex for a long time. The majority of men believed in this and gave it as a reason why they did not recommend their wives use the LARCs. About 20% of implant users experience amenorrhea, while up to 50% experience intermittent, regular, or protracted vaginal bleeding [40]. According to available literature, users of Implanon, one of the contraceptive implants (LARC), are more likely to experience infrequent or no menstrual bleeding than experience irregular bleeding, centrally to the perception that participants hold [41]. Although prolonged vaginal bleeding is a known side effect of some LARCs, it can be well managed by service providers if the women experiencing it visit health facilities [39, 42]. However, when men perceive LARCs as a method that will starve them sexually, the majority cannot accept their wives using such a method. This could be the reason for the low use of LARCs in the two regions in this study.

### Low libido

Loss of libido in women is linked to the use of Depot medroxyprogesterone acetate (DMPA) implants and vaginal rings, but not hormonal Intrauterine Devices (IUDs) [43]. The men’s concern over libido loss aligns with a Swedish study by Omar et al. [44], who expressed concern that contemporary contraceptives could affect future fertility. Due to these adverse effects, male companions find it undesirable for their women to use all LARCs, even if not all women experience this effect. There is, however, no evidence that men whose spouses use LARCs experience a loss of libido. Men may hold this perception due to their limited knowledge of LARCs [45]. Caruso et al. [46] discovered in their study that implant users had an increased desire to have sex, disproving the belief among participants that LARCs cause reduced libido in women. This is also corroborated by Guida et al. [47] in a study which revealed an enhancement in sexual activity among LARC users.

### Effect on the body organs

According to this study’s findings, indigenous Ugandan men indicated that implants, or IUDs, can disappear into the stomach and heart, leading to additional health issues such as malignancies, “pressure” (hypertension), and other complications in the body. However, this assertion is not supported by any available published literature. This finding could be due to the perception that men have that makes them not support the use of LARCs by their rural women. However, the finding agrees with the results from a study by Boivin, Carrier, Zulu, and Edwards [48], even though there is no published evidence that implants and IUDs, when used, can disappear in the body and cause more problems. The findings in the Rubanda and Kiboga districts are consistent with those done in Ethiopia [49], which found that participants feared using LARCs for a variety of reasons, including the disappearance of implants in the body and the perception that IUDs cause cancer and harm to the genitalia. Some participants feared that LARCs could destroy the “eggs” of women, rendering them incapable of feature reproduction. This finding is consistent with a study by Boivin et al [48], which found that men have similar concerns about contraceptives scorching the embryo. However, this assertion by men is a perception, as no literature supports the claim. The men’s reluctance to support their wives’ use of LARCs is due to the incorrect information they possess about those methods [48–50].

### Infertility of women

Similar to research conducted in Malawi [51] that confirmed the fear of side effects as an impediment to the adoption of Implants and IUDs, the current study demonstrated that fear of side effects is a barrier to the adoption of Implants and IUDs. Fear of infertility and delayed conception is consistent with the findings of a study conducted in Ethiopia [52] that discovered the husband’s support for contraception was closely related to the belief that they cause infertility, as well as the findings of a study conducted in Uganda [50] that found men were concerned about infertility caused by IUDs.

As reversible contraceptives, implants and IUDs do not cause infertility or delay the return to fertility after cessation of use, according to the available literature [41]. Since many men seem not to understand the reversibility of LARCs, this could explain why they are against their wives utilising the same methods of contraception. This is validated by two studies [53] and [38], which found that the non-use of LARCs by women was due to fear of decreasing future fertility and damaging the body through the same method.

The present study’s findings concur with [54], a Ugandan study that confirmed that contraceptive use was lower among women who believed the methods affected future fertility. Similar conclusions were reached in studies conducted in California and Kenya [55]; [39], which confirmed that participants’ concerns about infertility are significant barriers to their use. Similarly, a study conducted in China [56] discovered that the fear of future infertility was a significant barrier to IUDs adoption. However, studies have shown that some hormonal LARCs may lead to a delay in return to fertility up to one year, which can be misinterpreted by the population as infertility [57, 58].

### Domestic violence, separation of couples and single mothers

This finding is consistent with one study conducted in Ethiopia [52], which found that women feared using contraceptives because they believed that if they became infertile, their spouses would leave them. This contrasts the current study’s findings, which indicate that men fear their spouses will abandon them for other men. Similarly, a study conducted in Nigeria found that some males force contraceptive-using women to leave their residences, resulting in separation [42]. Separation was also detected in a second Kenyan study [39] that identified the same concerns among men.

### Challenges with removal or management of side effects

As a result of the lack of removal services and management of side effects for LARCs, men indicated spending a lot of money to have their women get managed for side effects and removal of the devices. This made the participants discourage their wives from using the LARCs. This finding is related to a study by Adeagbo et al. [58] in South Africa, which found that nurses lacked experience with Implanon NXT removals, leading to low uptake of the same method. The lack of LARCs removal services was also verified by women in a study done in Kenya [59]. This, therefore, could mean that men’s fears are shared by their women, and all contribute to the low uptake of LARCs. It can be deduced that this practice also contributes to the low uptake of LARCs unless the healthcare providers at the health facilities are well-trained in the recommended method.

### LARCs use leads to adultery

This study’s findings regarding men’s fear of adultery are consistent with Kenya’s studies [61], in which participants were afraid to use contraceptives because they believed their partners would suspect them of infidelity. In the same study, women feared that using contraceptives would encourage their male companions to engage in extramarital relations. In his research, Mwaisaka et al. [39] also identified fear of adultery as a concern for the use of contraceptives.

Therefore, participants in the current study hesitated to permit their wives to use LARCs to prevent infidelity in their households. In addition to findings from the Rubanda and Kiboga Districts, a study [61] found that permitting women to use contraceptives would make them healthier, more appealing, and more desirable to men. Due to this conviction, men in the districts of Rubanda and Kiboga cannot permit their spouses to use LARCs.

### Fear of losing their land to other tribes

No literature from Ugandan or international studies supports this finding in the Kiboga district. This fear might be a widespread myth from tribal distrust and prejudice and the experiences of land acquisition by people from other tribes in the district.

### LARC use leads to producing children with disability

The fear of having children with disabilities due to the use of LARCs is supported by research conducted in Tanzania [62]. Similarly, the findings of the study conducted in Kenya [39] corroborated the notion that contraceptives may cause birth defects in children.

### Complications in subsequent deliveries

The available literature shows that it is not true that women who use LARCs get complications in their subsequent pregnancies [12]. This finding from the current study concurs with a study in Uganda by [49] in which men were concerned that IUDs could cause future complications in subsequent pregnancies and birth. This also relates to the findings of a study done in Kenya which verified that men feared that using IUDs complicated deliveries among their partners [39]. The conclusions of the current research in Rubanda and Kiboga Districts confirm those from another study done in Uganda [63], where participants indicated that contraceptive use could cause the many caesarian sections in their region. However, no literature shows that contraceptive use leads to complications. This perception could be related to the limited information the men have regarding LARCs. When men have misinformation and little details on LARCs, they are more likely to oppose their use by their female partners [44].

### Study recommendations

The study recommends strengthening social and behavioural change communication through re-orientation and deployment of village health teams and community and religious leaders to sensitise the communities on the benefits of LARCs and address the negative perceptions of men on LARCs. The district health management teams of Rubanda and Kiboga, working with the health facility staff and supported by the implementing partners, should identify, train, and deploy male LARCs satisfied users as champions to disseminate positive and correct information about LARCs. By the champions sharing their positive experiences regarding LARC use in the communities, they could increase the knowledge and understanding of LARCs among men, changing their negative perceptions. The district health management teams of Rubanda and Kiboga, working with the implementing partners, should strengthen service provision, monitoring and evaluation systems for LARCs to address the gaps, such as managing side effects and removal services. Additionally, policymakers should provide a conducive environment for LARCs services where all services, including insertion, removals, and management of side effects, are effectively offered in all health facilities.

### Limitations of the study

Despite the study’s empiric findings on the perceptions of Ugandan men regarding the use of LARCs by their rural women, the study has some limitations. The study had a limited sample size and was conducted in a specific geographical area. However, the researcher overcame this limitation by having a more representative sample of 61 participants. Moreover, conducting the study in two distant districts and sub-counties in each district adds improved validity to the study that could be applied to similar contexts. The qualitative nature of this research is subjective and cannot measure how the perceptions influence men’s opposition to LARC use among rural women. This limitation was overcome by recommending more quantitative studies to determine how the perceptions of men influence their opposition to LARCs use. The qualitative research design meant that the study documented the experiences of a relatively small number of participants, which has negative consequences for the generalizability of the study’s findings to other contexts. Due to the context-specific nature of the qualitative study, the analysis and interpretation of the research data were heavily reliant on the researcher’s decisions; however, the same data could have been interpreted differently by another researcher, which could have led to different conclusions.

Despite the limitations above, the results are reliable, valid, and trustworthy. This is especially true because the emic methods used to collect and analyse the data were thorough and were checked by an expert in qualitative research at every step of the data collection, analysis, and reporting process. Even though there are limitations, the results show strong evidence that indigenous Ugandan men have negative perceptions about using LARCs. As a result, the men dissuade the rural women from using those contraceptive methods, justifying the need to mobilise their support for LARC use, which this study addresses.

## Conclusion

The study concluded that negative perceptions of indigenous men have made them not support the use of LARCs by rural women. To address the negative perceptions, the proposed recommendations above need to be implemented with the support of the Ministry of Health, Rubanda and Kiboga district local governments, community leaders, and health care providers. If the recommendations of this study are adopted and implemented, the knowledge and understanding of the benefits of LARCs among men and the general community could increase, thereby addressing the negative perceptions men have about LARCs. This would eventually result in more men having positive perceptions and being more likely to support rural women’s use of LARCs, which could lead to increased utilisation of those methods. The increased use of LARCs by rural women would, in the long run, result in reduced unintended pregnancies and their effects that contribute significantly to maternal morbidities and mortalities in Rubanda and Kiboga districts, bringing the benefits of using LARCs to the family, community, and country.

### Electronic supplementary material

Below is the link to the electronic supplementary material.

Supplementary Material 1

## Data Availability

The primary study document contains all the necessary detailed information and data set used and analysed during this study. It is available upon an appropriate request from the corresponding author.

## References

[1] Bain LE, Zweekhorst MB, de Cock Buning T (2020). Prevalence and determinants of unintended pregnancy in sub–saharan Africa: a systematic review. Afr J Reprod Health.

[2] Alene M, Yismaw L, Berelie Y, Kassie B, Yeshambel R, Assemie MA (2020). Prevalence and determinants of unintended pregnancy in Ethiopia: a systematic review and meta-analysis of observational studies. PLoS ONE.

[3] Gharaee M, Baradaran HR (2020). Consequences of unintended pregnancy on mother and fetus and newborn in North-East of Iran. J Maternal-Fetal Neonatal Med.

[4] World Health Organization. Trends in maternal mortality 2000 to 2017: estimates by WHO, UNICEF, UNFPA, World Bank Group and the United Nations Population Division: executive summary. World Health Organization; 2019.

[5] Bearak J, Popinchalk A, Ganatra B, Moller AB, Tunçalp Ö, Beavin C, Kwok L, Alkema L (2020). Unintended pregnancy and abortion by income, region, and the legal status of abortion: estimates from a comprehensive model for 1990–2019. The Lancet Global Health.

[6] Mohamed EA, Hamed AF, Yousef F, Ahmed EA (2019). Prevalence, determinants, and outcomes of unintended pregnancy in Sohag district. Egypt J Egypt Public Health Association.

[7] Bellizzi S, Pichierri G, Menchini L, Barry J, Sotgiu G, Bassat Q. The impact of underuse of modern methods of contraception among adolescents with unintended pregnancies in 12 low-and middle-income countries. J Global Health. 2019;9(2). 10.7189/jogh.09.020429 PMC6815657 31673342

[8] Bellizzi S, Mannava P, Nagai M, Sobel HL (2020). Reasons for discontinuation of contraception among women with a current unintended pregnancy in 36 low and middle-income countries. Contraception.

[9] UNICEF, UNFPA, World Bank Group and UNDESA/Population Division (2023). Mortality 2000 to 2020: estimates by WHO.

[10] Ministry of Health Uganda (2021). The Second National Family planning costed implementation plan 2020/21- 2024/25 (FP-CIP II).

[11] Safe Abortion Action Fund. Making unsafe abortion history in Uganda. [Online] Available at: https://saafund.org/making-unsafe-abortion-history-in Uganda; 2022[Accessed 11th July 2023].

[12] WHO/RHR & CCP (2018). Family Planning: A Global Handbook for Providers.

[13] Inzama W, Kaye DK, Kayondo SP, Nsanja JP (2023). Gaps in available published data on abortion in Uganda and the missed opportunity to inform policy and practice. Int J Gynecol Obstet.

[14] Uganda Bureau of Statistics (2017). The National Population and Housing Census 2014 – health status and Associated factors.

[15] Kakande NP, Galande J, Makombe R, Nyegenye W, Basaala AS, Mutyaba D (2019). Uganda Family Planning Atlas.

[16] World Health Organisation (2015). Strategies toward ending preventable maternal mortality (EPMM).

[17] FP2020. Family Planning 2020. [Online] Available at: https://www.familyplanning2020.org/sites/default/files/Uganda%202020%201-9%20Handout.pdf [Accessed 11th August 2021].

[18] Lugobe HM, Boatin AA, Asiimwe F, Karungi C, Kayondo M, Mukiza C, Wasswa S, Ngonzi J, Wylie BJ, Tamwesigire I (2021). 490 maternal mortality at a referral hospital in southwestern Uganda: a 5 year descriptive analysis. Am J Obstet Gynecol.

[19] Uganda Bureau of Statistics (2020). Family Planning Atlas 2020.

[20] Kakande NP, Galande J, Makombe R, Nyegenye W, Basaala (2019). AS & Mutyaba,D. Uganda Family Planning Atlas.

[21] Muheirwe F, Nuhu S (2019). Men’s participation in maternal and child health care in western Uganda: perspectives from the community. BMC Public Health.

[22] Sarfraz M, Hamid S, Kulane A, Jayasuriya R (2023). The wife should do as her husband advises’: understanding factors influencing contraceptive use decision making among married pakistani couples—qualitative study. PLoS ONE.

[23] Willcox M, King E, Fall E, Mubangizi V, Nkalubo J, Natukunda S, Nahabwe H, Goodhart C, Graffy J (2019). Barriers to uptake of postpartum long-acting reversible contraception: qualitative study of the perspectives of ugandan health workers and potential clients. Stud Fam Plann.

[24] Andardi B, Rahim DG, Achadi A (2022). Reasons of Refusal to Long-Acting Reversible Contraception (LARC) on Reproductive Age Women: a scoping review. e-CliniC.

[25] Leavy P (2017). Research Design: quantitative, qualitative, mixed methods, Arts-Based, and Community-Based Participatory Research Approaches.

[26] FP2020. Core Indicator Summary Sheet: 2019–2020 Annual Progress Report. 2020. Available at: https://fp2030.org/sites/default/files/Uganda%202020%20CI%20Handout.pdf [Accessed 17th November 2021].

[27] Uganda Bureau of Statistics (UBOS) and ICF (2018). Uganda Demographic and Health Survey 2016, Kampala, Uganda and Rockville.

[28] Hinton L, Ryan S, Interviews. In Pope C, Mays N, editors. Qualitative Research in Health Care. Fourth edition. Oxford: John Wiley & Sons Ltd; 2020;45–55.

[29] Campbell S, Greenwood M, Prior S, Shearer T, Walkem K, Young S, Bywaters D, Walker K (2020). Purposive sampling: complex or simple? Research case examples. J Res Nurs.

[30] Walliman N. Collecting Primary Data. Research Methods: The Basics. 2nd edition. New York: Routledge; 2018;141–173.

[31] Sekaran U, Bougie R (2016). Research Methods for Business: A Skill-Building Approach.

[32] McGrath C, Palmgren PJ, Liljedahl M (2019). Twelve tips for conducting qualitative research interviews. Med Teach.

[33] Cohen L, Manion L, Morrison K (2018). Research Methods in Education.

[34] Siedlecki SL (2022). Conducting interviews for qualitative Research Studies. Clin Nurse Specialist.

[35] Lester JN, Cho Y, Lochmiller CR (2020). Learning to do qualitative data analysis: a starting point. Hum Resour Dev Rev.

[36] Raskind IG, Shelton RC, Comeau DL, Cooper HL, Griffith DM, Kegler MC (2019). A review of qualitative data analysis practices in health education and health behavior research. Health Educ Behav.

[37] Mezmir EA (2020). Qualitative data analysis: an overview of data reduction, data display, and interpretation. Res Humanit Social Sci.

[38] Luo Z, Gao L, Anguzu R, Zhao J (2018). Long-acting reversible contraceptive use in the post-abortion period among women seeking abortion in mainland China: intentions and barriers. Reproductive Health.

[39] Mwaisaka J, Gonsalves L, Thiongo M, Waithaka M, Sidha H, Agwanda A, Mukiira C, Gichangi P (2020). Exploring contraception myths and misconceptions among young men and women in Kwale County, Kenya. BMC Public Health.

[40] Melville C (2015). Sexual and Reproductive Health at a glance.

[41] Akamike IC, Madubueze UC, Okedo-Alex IN, Anyigor CJ, Azuogu BN, Umeokonkwo CD, Mbachu CO (2020). Perception, pattern of use, partner support and determinants of uptake of family planning methods among women in rural communities in Southeast Nigeria. Contracept Reproductive Med.

[42] Boozalis MA, Tutlam NT, Robbins CC, Peipert JF (2016). Sexual desire and hormonal contraception. Obstet Gynecol.

[43] Omar B, Larsson EC, Calza S, Osman F (2022). Perceptions of family planning among some somali men living in Sweden: a phenomenographic study. Sex Reproductive Healthc.

[44] Kriel Y, Milford C, Cordero J, Suleman F, Beksinska M, Steyn P, Smit JA (2019). Male partner influence on family planning and contraceptive use: perspectives from community members and healthcare providers in KwaZulu-Natal, South Africa. Reproductive Health.

[45] Caruso S, Palermo G, Caruso G, Rapisarda AM (2022). How does contraceptive use affect women’s sexuality? A novel look at sexual acceptability. J Clin Med.

[46] Guida M, Farris M, Aquino CI, Rosato E, Cipullo L, Bastianelli C. Nexplanon subdermal implant: assessment of sexual profile, metabolism, and bleeding in a cohort of Italian women. BioMed Research International. 2019. 10.1155/2019/3726957 PMC6374865 30834263

[47] Boivin J, Carrier J, Zulu JM, Edwards D (2020). A rapid scoping review of fear of infertility in Africa. Reproductive Health.

[48] Endriyas M, Eshete A, Mekonnen E, Misganaw T, Shiferaw M (2018). Where we should focus? Myths and misconceptions of long-acting contraceptives in Southern Nations, Nationalities and People’s Region, Ethiopia: qualitative study. BMC Pregnancy Childbirth.

[49] Thummalachetty N, Mathur S, Mullinax M, DeCosta K, Nakyanjo N, Lutalo T, Brahmbhatt H, Santelli JS (2017). Contraceptive knowledge, perceptions, and concerns among men in Uganda. BMC Public Health.

[50] Tebeje B, Workneh D (2017). Prevalence, perceptions and factors contributing to long-acting reversible contraception use among family planning clients, Jimma Town, Oromiya Region, South-West Ethiopia. J Women’s Health Care.

[51] Sedlander E, Yilma H, Emaway D, Rimal RN (2022). If fear of infertility restricts contraception use, what do we know about this fear? An examination in rural Ethiopia. Reproductive Health.

[52] Damayanti R, Nisa H, Ariawan I, Titaley C, Dachlia D, Wahyuningrum Y, Storey D (2019). Why don’t couples use the contraceptive that’s best for them? Social determinants of long acting and permanent contraceptive method use in Indonesia. Indian J Public Health Res Dev.

[53] Zimmerman LA, Sarnak DO, Karp C, Wood SN, Moreau C, Kibira SP, Makumbi F (2021). Family planning beliefs and their association with contraceptive use dynamics: results from a longitudinal study in Uganda. Stud Fam Plann.

[54] Cabral MA, Schroeder R, Armstrong EM, El Ayadi AM, Gürel AL, Chang J, Harper CC (2018). Pregnancy intentions, contraceptive knowledge and educational aspirations among community college students. Perspect Sex Reprod Health.

[55] Feng X, Shi S (2022). Intrauterine Contraception Use among Women receiving Post-Abortion Care in Guangzhou, China: a cross-sectional study. Clin Exp Obstet Gynecol.

[56] Barden-O’Fallon J, Speizer IS, Calhoun LM (2021). Return to pregnancy after contraceptive discontinuation to become pregnant: a pooled analysis of West and East African populations. Reprod Health.

[57] Gayatri M, Utomo B, Budiharsana M, Dasvarma G. Pregnancy resumption following contraceptive discontinuation. Hazard survival analysis of the Indonesia Demographic and Health Survey Data; 2007. 10.1371/journal.pone.0264318 PMC8865679 35196329

[58] Adeagbo OA, Mullick S, Pillay D, Chersich MF, Morroni C, Naidoo N, Pleaner M, Rees H. Uptake and early removals of Implanon NXT in South Africa: perceptions and attitudes of healthcare workers. South Afr Med J. 2017;107(10). 10.7196/SAMJ.2017.v107i10.12821 29397681

[59] Britton LE, Williams CR, Onyango D, Wambua D, Tumlinson K (2021). When it comes to time of removal, nothing is straightforward: a qualitative study of experiences with barriers to removal of long-acting reversible contraception in western Kenya. Contraception: X.

[60] Obare F, Odwe G, Cleland J (2021). Men’s needs and women’s fears: gender-related power dynamics in contraceptive use and coping with consequences in a rural setting in Kenya. Cult Health Sex.

[61] Bekele D, Surur F, Nigatu B, Teklu A, Getinet T, Kassa M, Gebremedhin M, Gebremichael B, Abesha Y (2021). Contraceptive prevalence rate and associated factors among reproductive age women in four emerging regions of Ethiopia: a mixed method study. Contracept Reproductive Med.

[62] Kassim M, Ndumbaro F (2022). Factors affecting family planning literacy among women of childbearing age in the rural Lake zone, Tanzania. BMC Public Health.

[63] Waniala I, Nakiseka S, Nambi W, Naminya I, Osuban Ajeni M, Iramiot J, Nekaka R, Nteziyaremye J (2020). Prevalence, indications, and community perceptions of caesarean section delivery in Ngora District, Eastern Uganda: mixed method study. Obstet Gynecol Int.

