# Supplementary material for: Perceptions of indigenous ugandan men on the use of long acting reversible contraceptives (LARCs) by rural women
Source: Contracept Reprod Med. 2023 Oct 16;8:50. doi: 10.1186/s40834-023-00246-8 (PMC10577931; doi:10.1186/s40834-023-00246-8)
Supplement: Supplementary file 1 — Supplementary Material 1 [file 40834_2023_246_MOESM1_ESM.docx]

**BIODATA OF THE PARTICIPANTS OF THE STUDY DONE IN RUBANDA AND KIBOGA DISTRICTS OF UGANDA**

| **No.** | **Age** | **Occupation** | **Language** | **Village** | **Parish** | **Sub-county** | **Tribe** | **Marital status** | **No of years spent in district** | **No. of children** |
| --- | --- | --- | --- | --- | --- | --- | --- | --- | --- | --- |
| **Kiboga district** | | | | | | | | | | |
| FGD K1 | 38 | Farmer | Luganda | Temanakali A | Matagi | Bukomero | Muganda | Married | 11 | 6 |
| FGD K2 | 30 | Mechanic | Luganda | Temanakali A | Matagi | Bukomero | Muganda | Married | 18 | 2 |
| FGD K3 | 20 | Farmer | Luganda | Temanakali A | Matagi | Bukomero | Munyoro | Married | 20 | 2 |
| FGD K4 | 25 | Farmer | Luganda | Temanakali A | Matagi | Bukomero | Muganda | Married | 25 | 1 |
| FGD K5 | 30 | Motorcyclist | Luganda | Temanakali A | Matagi | Bukomero | Muganda | Married | 30 | 4 |
| FGD K6 | 32 | Farmer | Luganda | Temanakali A | Matagi | Bukomero | Muganda | Married | 32 | 2 |
| FGD K7 | 28 | Barber | Luganda | Temanakali A | Matagi | Bukomero | Muganda | Married | 13 | 2 |
| FGD K8 | 28 | Motorcyclist | Luganda | Temanakali A | Matagi | Bukomero | Muganda | Married | 28 | 3 |
| FGD K9 | 29 | Motorcyclist | Luganda | Temanakali A | Matagi | Bukomero | Muganda | Married | 29 | 3 |
| FGD K10 | 55 | Farmer | Luganda | Temanakali A | Matagi | Bukomero | Muganda | Married | 55 | 13 |
|  | | | | | | | | | | |
| I K 11 | 46 | Farmer | Luganda | Temanakali A | Matagi | Bukomero | Muganda | Married | 46 | 6 |
| I K 12 | 39 | Farmer | Luganda | Temanakali A | Matagi | Bukomero | Muganda | Married | 39 | 5 |
| I K 13 | 44 | Farmer | Luganda | Temanakali A | Matagi | Bukomero | Muganda | Married | 44 | 10 |
| I K 14 | 23 | Farmer | Luganda | Temanakali A | Matagi | Bukomero | Muganda | Married | 23 | 0 |
| I K 15 | 32 | Farmer | Luganda | Temanakali A | Matagi | Bukomero | Muganda | Married | 32 | 4 |
|  | | | | | | | | | | |
| FGD KD 1 | 23 | Motorcyclist | Luganda | Muyenje | Lwankonge | Dwaniro | Muganda | Married | 7 | 1 |
| FGD KD 2 | 37 | Farmer | Luganda | Muyenje | Lwankonge | Dwaniro | Muganda | Married | 10 | 2 |
| FGD KD 3 | 25 | Farmer | Luganda | Muyenje | Lwankonge | Dwaniro | Muganda | Married | 7 | 2 |
| FGD KD 4 | 40 | Farmer | Luganda | Muyenje | Lwankonge | Dwaniro | Muganda | Married | 40 | 8 |
| FGD KD 5 | 25 | Farmer | Lusoga | Muyenje | Lwankonge | Dwaniro | Musoga | Married | 20 | 1 |
| FGD KD 6 | 43 | Farmer | Luganda | Muyenje | Lwankonge | Dwaniro | Muganda | Married | 17 | 3 |
| FGD KD 7 | 36 | Farmer | Luganda | Muyenje | Lwankonge | Dwaniro | Muganda | Married | 36 | 4 |
| FGD KD 8 | 43 | Motorcyclist | Lusoga | Muyenje | Lwankonge | Dwaniro | Muteso | Married | 17 | 7 |
| FGD KD 9 | 33 | Farmer | Luganda | Muyenje | Lwankonge | Dwaniro | Muganda | Married | 33 | 5 |
| FGD KD 10 | 38 | Farmer | Rutooro | Muyenje | Lwankonge | Dwaniro | Mutooro | Married | 8 | 1 |
|  | | | | | | | | | | |
| I KD 11 | 25 | Farmer | Luganda | Muyenje | Lwankonge | Dwaniro | Munyankore | Married | 25 | 2 |
| I KD 12 | 29 | Motorcyclist | Luganda | Muyenje | Lwankonge | Dwaniro | Muganda | Married | 10 | 2 |
| I KD 13 | 22 | Farmer | Kinyarwanda | Muyenje | Lwankonge | Dwaniro | Rwandan | Married | 5 | 1 |
| I KD 14 | 20 | Motorcyclist | Luganda | Muyenje | Lwankonge | Dwaniro | Muganda | Married | 20 | 1 |
| I KD 15 | 31 | Beekeeper | Luganda | Muyenje | Lwankonge | Dwaniro | Muganda | Married | 21 | 4 |
| FGD R1 | 42 | Farmer | Rukiga | Rushaki | Nangara | Nyamweru | Mukiga | Married | 42 | 5 |
| FGD R 2 | 47 | Farmer | Rukiga | Rushaki | Nangara | Nyamweru | Mukiga | Married | 47 | 5 |
| FGD R3 | 27 | Farmer | Rukiga | Kihengamo | Nangara | Nyamweru | Mukiga | Married | 27 | 2 |
| FGD R4 | 34 | Farmer | Rukiga | Kihorongwa | Nangara | Nyamweru | Mukiga | Married | 34 | 3 |
| FGD R5 | 38 | Businesman | Rukiga | Kakamisa | Muyenje | Bubare | Mukiga | Married | 38 | 2 |
| FGD R6 | 35 | Motorcyclist | Rukiga | Kihengamo | Nangara | Nyamweru | Mukiga | Married | 35 | 3 |
| FGD R7 | 43 | Farmer | Rukiga | Kagana cell | Nangara | Nyamweru | Mukiga | Married | 43 | 5 |
| FGD R8 | 48 | Motorcyclist | Rukiga | Kagugo | Bwayu | Nyamweru | Mukiga | Married | 48 | 9 |
| FGD R9 | 48 | Business | Rukiga | Kagana cell | Nangara | Nyamweru | Mukiga | Married | 48 | 12 |
| FGD R10 | 45 | Farmer | Rukiga | Rujanjara | Bwayu | Nyamweru | Mukiga | Married | 45 | 7 |
|  | | | | | | | | | | |
| I R 11 | 34 | Motorcyclist | Rukiga | Kihengamo | Nangara | Nyamweru | Mukiga | Married | 34 | 3 |
| I R12 | 36 | Motorcyclist | Rukiga | Kagana cell | Nangara | Nyamweru | Mukiga | Married | 36 | 2 |
| I R13 | 48 | Farmer | Rukiga | Kihengamo | Nangara | Nyamweru | Mukiga | Married | 48 | 5 |
| I R14 | 31 | Farmer | Rukiga | Rushaki | Nangara | Nyamweru | Mukiga | Married | 31 | 4 |
| I R15 | 32 | Farmer | Rukiga | Bicerere | Nyarurambi | Nyamweru | Mukiga | Married | 32 | 2 |
| RM 1 | 42 | Teacher | Rukiga | Rushambya | Nyarurambi | Muko | Mukiga | Married | 42 | 4 |
| RM 2 | 38 | Farmer | Rukiga | Katasya | Nyarurambi | Muko | Mukiga | Married | 38 | 1 |
| RM 3 | 27 | Farmer | Rukiga | Rwamabale | Nyarurambi | Muko | Mukiga | Married | 27 | 1 |
| RM 4 | 46 | Farmer | Rukiga | Rushambya | Nyarurambi | Muko | Mukiga | Married | 46 | 6 |
| RM 4 | 25 | Farmer | Rukiga | Bugunga | Nyarurambi | Muko | Mukiga | Married | 25 | 2 |
| RM 6 | 52 | Farmer | Rukiga | Katasya | Nyarurambi | Muko | Mukiga | Married | 52 | 6 |
| RM 7 | 37 | Farmer | Rukiga | Bicerere | Nyarurambi | Muko | Mukiga | Married | 37 | 5 |
| RM 8 | 33 | Farmer | Rukiga | Bicerere | Nyarurambi | Muko | Mukiga | Married | 33 | 4 |
| RM 9 | 44 | Farmer | Rukiga | Rwamabare | Nyarurambi | Muko | Mukiga | Married | 44 | 3 |
| RM10 | 49 | Farmer | Rukiga | Bucerere | Nyarurambi | Muko | Mukiga | Married | 49 | 9 |
|  | | | | | | | | | | |
| I RM 11 | 46 | Businessman | Rukiga | Rushanga | Nyarurambi | Muko | Mukiga | Married | 46 | 6 |
| I RM 12 | 25 | Farmer | Rukiga | Bugunga | Nyarurambi | Muko | Mukiga | Married | 25 | 2 |
| I RM 13 | 48 | Farmer | Rukiga | Bugunga | Nyarurambi | Muko | Mukiga | Married | 48 | 4 |
| I RM14 | 37 | Farmer | Rukiga | Katasya | Nyarurambi | Muko | Mukiga | Married | 37 | 4 |
| I RM 15 | 48 | Farmer | Rukiga | Bicerere | Nyarurambi | Muko | Mukiga | Married | 48 | 5 |
| I RM 16 | 24 | Farmer | Rukiga | Bugunga | Nyarurambi | Muko | Mukiga | Married | 24 | 1 |
